# Spiders in Thailand (SIT) via spiderthailand.info: Thailand spider data retrieval system for geographical occurrence and photographic identification

**DOI:** 10.3897/BDJ.12.e118262

**Published:** 2024-04-29

**Authors:** Booppa Petcharad, Thanakron Into, Sasiporn Tongman, Niwan Wattanakitrungroj, Nutthaphol Dechpramualphol, Supet Jirakajohnkool, Tadsanai Jeenthong

**Affiliations:** 1 Thammasat University Rangsit Campus, Pathum Thani, Thailand Thammasat University Rangsit Campus Pathum Thani Thailand; 2 King Mongkut's University of Technology Thonburi, Bangkok, Thailand King Mongkut's University of Technology Thonburi Bangkok Thailand; 3 National Science Museum Thailand, Pathum Thani, Thailand National Science Museum Thailand Pathum Thani Thailand

**Keywords:** arthropod, arachnid, website, online platform, spider distribution, lists of species, Southeast Asia

## Abstract

**Background:**

High biodiversity in the tropics is good for ecosystem services; however, challenges in taxonomy and identification usually come from such high biodiversity. Spiders are no exception to the challenges. Identifying spiders in tropical places like Thailand is difficult and time consuming. To reduce the difficulty of identifying Thai spiders, a data retrieval system for geographical occurrence and photographic identification was conducted to deploy on an online platform, Spiders in Thailand (SIT) via the website “spiderthailand.info”. This allows professional arachnologists and amateur spider lovers to visit and check the geographical distribution of Thai spiders and to quickly access pictures for comparative photographic identification. To facilitate Thai spider identification, there were two parts, the database and the website, which are connected to each other. Data of Thai spiders were extracted from the World Spider Catalog to build a database comprising geographical occurrence and pictures of spider species in Thailand. The database was then linked with the website to display data.

**New information:**

The dataset of pictures and illustrations extracted from taxonomic literature of the World Spider Catalog were included in the database for connecting with the online platform, Spiders in Thailand (SIT) via the website “spiderthailand.info” which facilitated access to pictures and illustrations, expediting the identification of Thai spider specimens. Geographical occurrences of Thai spiders consisted of 1419 records belonging to 670 species of 228 genera and 50 families. Amongst those, 461 species from 133 genera of 41 families were distributed only in Thailand. Around Thailand, 756 geographical localities were reported for spider occurrences. From 76 provinces and one additional special administrative area (Bangkok), 58 provinces showed occurrence records of spiders and 18 provinces showed non-occurrence records. Those provinces of non-occurrence records of spiders were Amnat Charoen, Ang Thong, Bueng Kan, Chai Nat, Maha Sarakham, Mukdahan, Nakhon Phanom, Nong Bua Lam Phu, Nonthaburi, Phayao, Phichit, Phra Nakhon Si Ayutthaya, Samut Prakan, Samut Sakhon, Si Sa Ket, Sing Buri, Uthai Thani and Yasothon. Most spiders were reported from Chiang Mai Province.

## Introduction

Spiders are a group of animals which are highly diverse in several aspects amongst terrestrial invertebrates (Coddington and Levi 1991, Kotze et al. 2022). They are a prominent predator amongst invertebrate communities in the world with a large number of species, more than 50,000 species formally described (Agnarsson 2023, World Spider Catalog 2023). Based on extracted data from the World Spider Catalog (2024) with interpretation (Petcharad et al., unpublished data), it showed that the study of spiders in Thailand started in 1767, 257 years ago. The intensive description of spider species is composed of six periods, in 1793, 1837, 1887, 1995, 2001, 2005 and from 2011 until now. Those periods are different from each other: 44, 50 and 108 years before 1995, respectively. Less than 10 years of differences between the periods or even continuing study after 1995 are notable as it is almost the same time as the first launching of the World Spider Catalog website by Platnick (World Spider Catalog 2023). It was presumed that the online platform of the World Spider Catalog supports identification and taxonomic study of Thai spiders. Nevertheless, the study of Thai spider taxonomy is still limited. There are no taxonomic books or compiled literature for identifying spiders in Thailand. Thailand is situated in a biodiversity hotspot (Singh et al. 2021, Pomoim et al. 2022) where several groups of organisms are of high species richness. However, spiders have been ignored for study and survey causing a slow growth of taxonomic databases in comparison to other groups like plants, mammals, birds, reptiles, amphibians, butterflies, ants, beetles, and snails in Thailand (Tovaranonte et al. 2013, Tantipisanuh and Gale 2018, Trisurat et al. 2019, Singh et al. 2021, Pomoim et al. 2022). For spiders in Thailand, a small taxonomic dataset amongst their diverse appearance creates a large gap of difficulty in building an identification key of Thai spiders. Consequently, there has currently been no identification key, which causes difficulty for the study of spiders and is an obstruction for the growth of the Thai spider database. Tools for identifying organisms, such as dichotomous keys are accurate and useful in the case of constructing such keys, based on a complete database of each taxon from a specific area. Otherwise, the dichotomous keys are at risk of providing a wrong result in identification. However, dichotomous keys can be done after creating a complete database, whereas creating a complete database is supported by dichotomous keys or certain identification keys. Having few identification keys have caused a challenge to identify Thai spiders even at genus level, and it appears it will be a long time before having a complete database for obtaining such keys. The World Spider Catalog (2023) is a comprehensive and searchable catalog that centralises the spider taxonomy and provides access to literature for all the formally described taxa. The identification protocol to indicate species is to check family, genus and species from a large amount of literature and all taxa at global scale, checking paper by paper and comparing each picture from those papers with a specimen being identified under a microscope. Accordingly, spider identification is time consuming, even for experienced arachnologists. A great example of an easy access platform for spider identification is the Spiders of Europe website (Nentwig et al. 2024) which facilitates identifying European spiders. To fill this gap, few identification keys and a small taxonomic dataset of Spiders in Thailand, literature of Thai spiders was extracted from the World Spider Catalog (2023) and pictures of Thai species were compiled in this study to facilitate any users who would like to identify spiders by comparing voucher specimens with spider pictures from taxonomic literature. A photographic identification tool via an online platform was served to identify Thai spiders. Geographical occurrences of spider data were uploaded in the platform for not only supporting identification, but also helping further study and collection plan decisions. The website “spiderthailand.info” was deployed as an online platform with a non-commercial aim. Two main purposes of this study were to gather data of Thai spiders from taxonomic literature and to present the data via an online platform in order to serve as a photographic identification key and record of geographical occurrence in Thailand. The main target groups of this online platform are students and researchers. Hopefully, this website will contribute to the growth of academic knowledge in arachnology amongst Southeast Asian countries, especially in Thailand, which is a biodiversity hotspot.

## Project description

### Study area description

Our study aimed to aggregate occurrences of spiders in Thailand that were mainly reported in taxonomic literature.

**Data resources and integrations**: In the first step, the daily updated csv file storing worldwide spider records was downloaded from World Spider Catalog: WSC (https://wsc.nmbe.ch/dataresources) on 9 September 2020 which contained spiders’ information for almost 50,000 species (World Spider Catalog 2020). Then, data stored in a “distribution” field were filtered using some specific keywords related to spiders found in Thailand. Those keywords were as follows: Thailand, Asia and Pacific islands, Tropical Asia, Temperate Asia, Southeast Asia, Asia, as well as some countries such as China, Nepal, India, Myanmar, Laos, Vietnam, Malaysia (Peninsula) and Indonesia (Bali, Java). All records associated with these keywords were pulled and data in some related fields, i.e. species, genus, family, author, and year were focused on in order to prepare a Thailand spider list. In the second step, information in the Thailand spider list was used as keywords for retrieving additional information from various data resources including articles in academic journals and websites. These data from such documents were not only manually skimmed and verified by us, but also roughly gathered for our manual verification with the help of Artificial Intelligence (AI) technology tools like SciSpace (https://typeset.io) and ChatDox (https://www.chatdox.com) to speed-up spider data integration process. After finishing this step, the Thailand spider data table in the form of an excel file storing spider species occurrences in Thailand contained the following properties: author, publish year, province, district, locality, location, latitude, longitude, altitude (if any), sampling method (if any), habitat (if any), microhabitat (if any) and designate (if any). Finally, Python code blocks were created in Google’s Colaboratory tool to convert this excel file into a json file as the NoSQL (Not Only SQL) database file ready for developing the website to display information about species of spiders found in Thailand including a Thailand’s province and district map.

### Design description

The infrastructure of spiderthailand.info is based on a MongoDB database, which is a NoSQL database. It does not use the traditional relational database model. However, MongoDB stores data in documents similar to JSON objects. In this way, it makes MongoDB very flexible and scalable, because documents can contain any type of data and can be nested to any depth. The website of spiderthailand.info does not provide standardised APIs. Instead, it offers a web application written in JavaScript language using NextJs for experts’ data submission and a web portal for everyone’s read-only data public access. Additionally, the website of spiderthailand.info contains geospatial features. This allows us to not only query each spider species, i.e. based on their names in Thailand, but also search a specific district or province for spatial data visualisation.

### Funding

This work was supported by the Thailand Science Research and Innovation Fundamental Fund fiscal year 2023.

## Web location (URIs)

Homepage: https://spiderthailand.info

## Technical specification

Programming language: Back-end programming language: Go

Operational system: Linux

Interface language: Front-end platform: NextJs (JavaScript)

Service endpoint: https://spiderthailand.info

## Repository

Type: Git

Browse URI: https://github.com/tuscb/spider-th-public

## Usage licence

### Usage licence

Creative Commons Public Domain Waiver (CC-Zero)

## Implementation

### Implements specification

**Figure 1.**
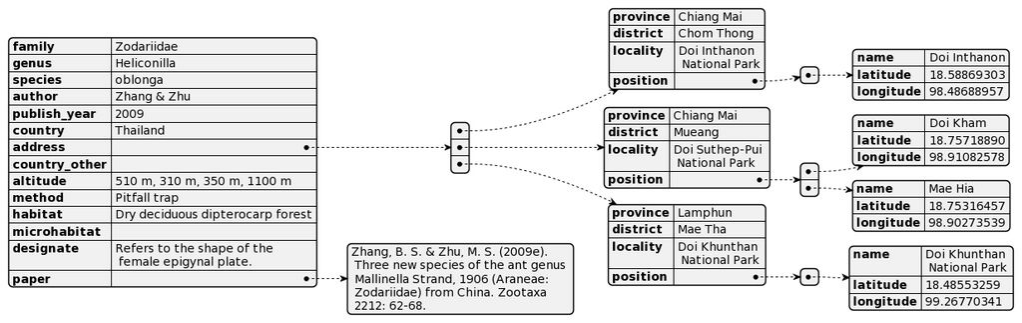
Example of one spider species, *Heliconillaoblonga* and its data stored in the spiderthailand.info database. Diagram was created by using https://plantuml.com.

**Data attribute description**:

Spider species’ occurrence data were represented by multiple attributes as explained in Table 1. The data were stored in our database as json structure. In Fig. 1, the example of one spider species’ information was depicted.

## Additional information

**Figure 2.**
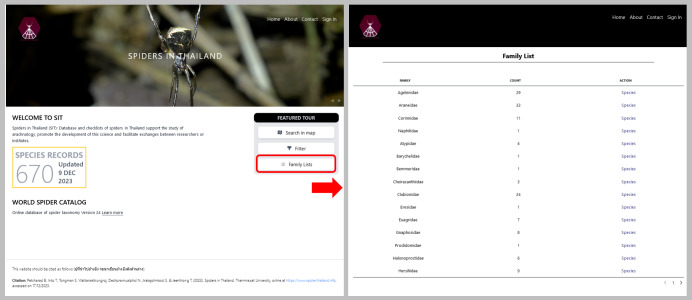
Front page of https://spiderthailand.info (on the left) and family list page (on the right).

**Figure 3.**
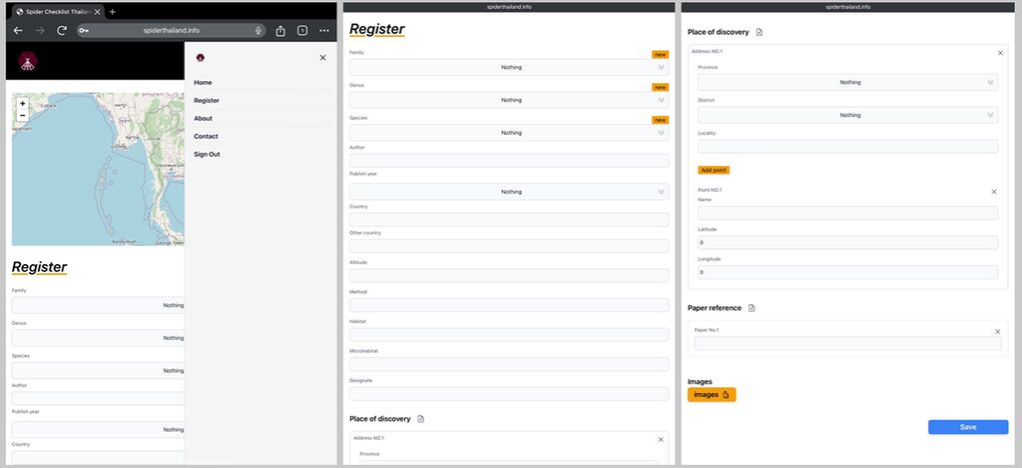
Register page accessed by an expert member from a mobile device in order to submit new updated data.

**Figure 4.**
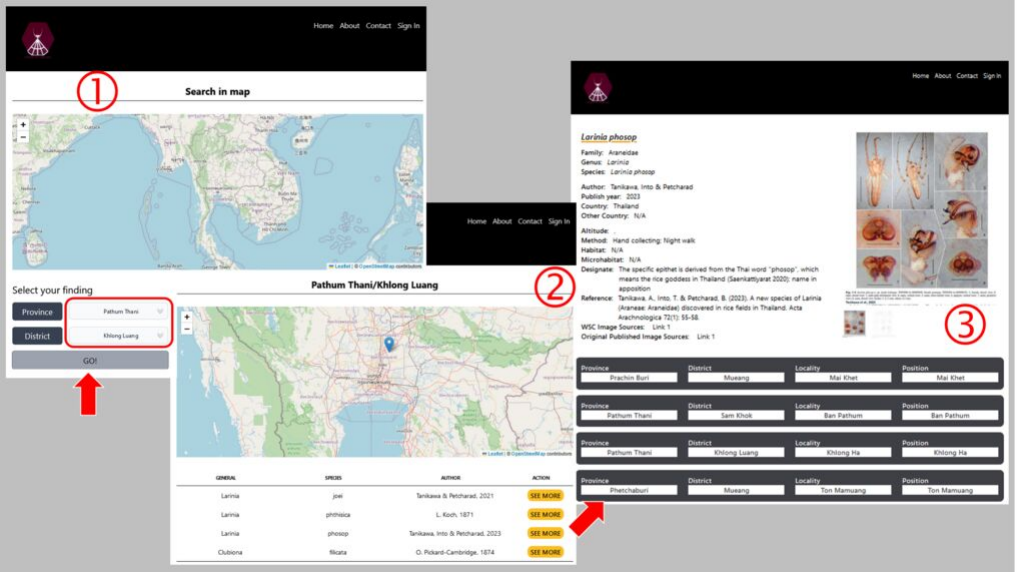
An example of querying spider's data in Thailand by choosing a province and a district.

**Figure 5.**
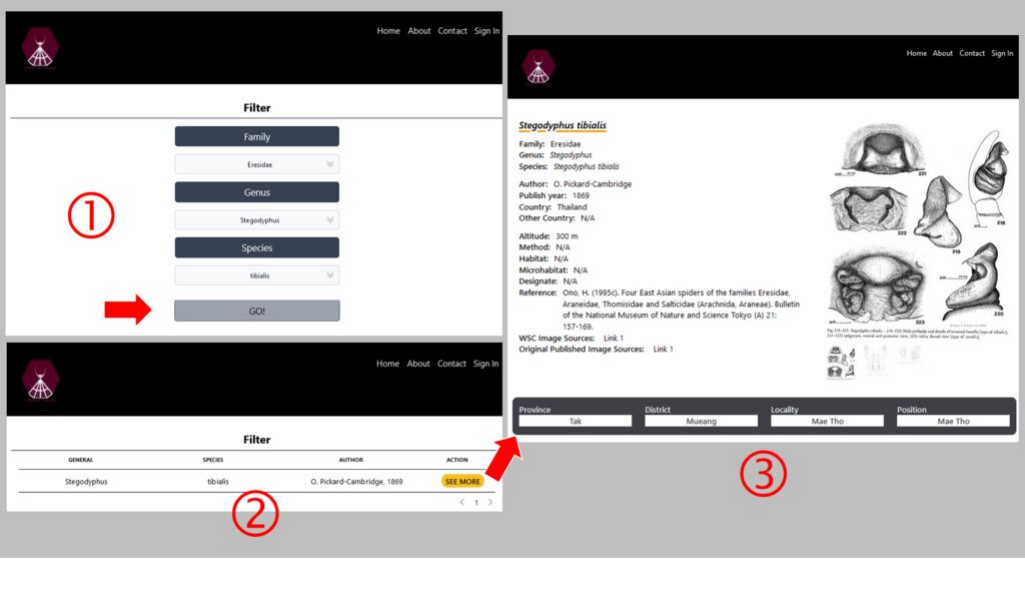
An example of querying spider's data in Thailand by indicating spider's taxonomic information such as family, genus, and species, respectively.

**Usage description**:

Users can always see the current total numbers of spider species found in Thailand on the main page (Fig. 2). The database of website, spiderthailand.info will be regularly updated by registered experts and main features of the website will be annually updated by the system maintainer. There is a register page for only sign-in accounts to fill-in new data (Fig. 3). In the FEATURE TOUR section of the main page, spiders and places can be searched by three main features. First, ‘Search in Map’ feature allows users to select a province and the district to find out if any spider species that have been reported there. The list of records as well as geographical locations on the map are shown and the details of each spider record including pictures can be viewed after clicking ‘SEE MORE’ as illustrated in Fig. 4. These pictures were provided for easy comparison with voucher specimens. Second, ‘Filter’ feature was provided for users to find each spider record via selecting family, genus and species as an example in Fig. 5. Third, occurrences of spiders in Thailand according to their family name were also summarised in the ‘Family Lists’ feature. Each family can be explored by clicking ‘Species’ (Fig. 2).

## Figures and Tables

**Table 1.** Field properies to store one data record.

Field Name	Data Type	Description
_id	object Id	ID of the spider.
spider_uuid	string	Unique ID of the spider.
family	string	Family of the spider.
genus	string	Genus of the spider.
species	string	Name of the spider.
author	string	Author of publications who firstly discovered the species.
publish_year	string	Year of publications about the species for the first time.
country	string	Thailand, as the first country to report spider occurrences.
country_other	string	Other countries reported spider occurrences.
altitude	string	The vertical distance of spider occurrences reported.
method	string	Procedure of spider occurrences reported.
habitat	string	Living areas of spider occurrences reported.
microhabitat	string	Sub-living areas of spider occurrences reported.
designate	string	Origin of spider’s species name.
status	string	State of data completion.
address[]	array of object	Nested data array stored area positions and names of spider occurrences reported.
address[].province	string	Province name.
address[].district	string	District name.
address[].locality	string	Location name of spider occurrences reported.
address[].position[]	array of object	Position of spider occurrences reported.
address[].position[].name	string	Name of each position.
address[].position[].latitude	float64	Latitude of a position.
address[].position[].longitude	float64	Longitude of a position.
image_file[]	array of string	Image file names.
paper[]	array of string	References to papers related to the occurrences.
create_at	date_time	Data and time of the first created record.
update_at	date_time	Data and time of the latest data modification.
created_by	string	User who submitted data.
